# Activity of Exebacase (CF-301) against Biofilms Formed by Staphylococcus epidermidis Strains Isolated from Prosthetic Joint Infections

**DOI:** 10.1128/aac.00588-22

**Published:** 2022-07-11

**Authors:** Aubin Souche, Camille Kolenda, Jordan Teoli, Raymond Schuch, Tristan Ferry, Frédéric Laurent, Jérôme Josse

**Affiliations:** a CIRI–Centre International de Recherche en Infectiologie, Inserm, U1111, CNRS UMR5308, ENS de Lyon, Université Claude Bernard Lyon 1, Lyon, France; b Laboratoire de Bactériologie, Institut des Agents Infectieux, Hospices Civils de Lyon, Lyon, France; c ContraFect Corporation, Yonkers, New York, USA; d Université Claude Bernard Lyon 1, Lyon, France; e Centre de Référence des Infections Ostéo-articulaires Complexes (CRIOAc Lyon), Hospices Civils de Lyon, Lyon, France; f Service des Maladies Infectieuses, Hospices Civils de Lyon, Lyon, France

**Keywords:** CF-301, biofilms, exebacase, prosthetic joint infections, *Staphylococcus epidermidis*

## Abstract

Staphylococcus epidermidis is one of the main pathogens responsible for bone and joint infections, especially those involving prosthetic materials, due to its ability to form biofilms. In these cases, biofilm formation, combined with increased antimicrobial resistance, often results in therapeutic failures. In this context, the development of innovative therapies active against S. epidermidis is a priority. The aim of this study was to evaluate the *in vitro* activity of the lysin exebacase (CF-301) against biofilms formed by 19 S. epidermidis clinical strains isolated from prosthetic joint infections (PJI). We determined the biomass and the remaining viable bacteria inside biofilms after 24 h of exposure to exebacase. Exebacase activity was compared to that of rifampicin, vancomycin, and daptomycin. The use of exebacase in addition to antibiotics was also assessed. Exebacase displayed (i) a significant anti-biomass activity on S. epidermidis biofilms at concentrations ≥5 mg/L (mean decrease up to 66% at 150 mg/L), (ii) significant bactericidal activity on biofilms at concentrations ≥50 mg/L (mean decrease up to 1.7 log CFU at 150 mg/L), (iii) synergistic effects when used in addition to rifampicin, vancomycin, or daptomycin. The extent of these activities varied by isolate. Exebacase can be considered a promising therapy in addition to rifampicin, vancomycin, or daptomycin in the context of PJI. Further *in vitro* studies are needed to understand its mechanism of action on S. epidermidis biofilms and *in vivo* investigations are required to confirm these data.

## INTRODUCTION

Bone and joint infections (BJI) are severe infections comprising septic arthritis, osteomyelitis, and spondylodiscitis. The majority of BJI (65%) are native infections, with the remaining clinical forms being associated with the presence of prosthetic material (prosthetic joint infections [PJI]) (1). These infections threaten the functional performance and overall health of patients. They often result in long and repeated hospitalizations, prolonged antibiotic treatments, and are associated with significant individual and societal costs, notably related to a decrease in the quality of life and extended sick leaves (1–4). Staphylococci are the most prevalent genus involved in BJI and PJI. Staphylococcus aureus is responsible for 30% to 70% of BJI (1, 2, 5–7), while other staphylococcal species, mainly Staphylococcus epidermidis, are involved in 17% to 48% of BJI, and are even more frequent in PJI (5–8). S. epidermidis infections are classically nosocomial or related to health care, and secondary to the contamination/colonization of foreign materials such as catheters or prosthetic materials (prosthetic joints, prosthetic valves) (9).

One of the major factors of S. epidermidis prevalence and treatment failures in PJIs is its ability to form biofilm on biotic and abiotic surfaces (10). Biofilm, formed by a bacterial community organization within an extracellular matrix made of exo-polysaccharides (polysaccharide intracellular adhesin [PIA]), proteins, and bacterial extracellular DNA (9, 11), provides a protection against antibiotics, immune cells and mediators produced by the host (12, 13). Hence, biofilm formation is a clinical issue resulting in difficult-to-treat infections (9). In addition, S. epidermidis clinical isolates responsible for PJI often show antibiotic susceptibility profiles with a high incidence of resistance, notably against methicillin, fluoroquinolones, rifampicin, and aminoglycosides (9, 14). In this context, therapeutic failures are frequently observed, and the development of new therapeutic options is needed. The use of lysins is a promising option because of their activity against planktonic cells as well as cells within biofilms, and their synergistic activity in association with antibiotics (15, 16).

Lysins are peptidoglycan hydrolases that destroy bacteria by targeting their cell wall (17). The use of purified recombinant lysins results in rapid bactericidal activity against specific bacteria (18). Exebacase (CF-301; ContraFect Corporation, Yonkers, NY, USA), a lysin with anti-staphylococcal activity, is the most advanced lysin tested in clinical trials and is currently in phase III for treatment of S. aureus bacteremia/right-sided endocarditis (ClinicalTrials.gov Identifier: NCT04160468) (19). In addition to its activity against S. aureus (15, 16), exebacase exhibits *in vitro* activity against both planktonic and biofilm S. epidermidis (16, 20). It has been shown that exebacase activity is potentiated in the presence of albumin, the main protein in joint fluid (21). Currently, no resistance to exebacase has been described (15, 20) and exebacase has a significant postantibiotic effect (22). In addition, an effect on the production of specific virulence factors has been reported for exebacase, even at low concentrations, in particular for some involved in the agglutination and biofilm formation of S. aureus strains (22). *In vivo*, activity against methicillin-resistant S. aureus has been demonstrated in multiple models, including an osteomyelitis model in rats (23). Together, these data also support the study of exebacase to target biofilms in the context of S. epidermidis PJI. This potential therapeutic use of exebacase is further supported by the favorable outcomes reported with compassionate use of exebacase for patients with PJI caused by S. epidermidis and treated in France under individual Autorisation Temporaire D'utilisation (ATU) issued by the French National Agency for Medicines and Health Products Safety (ANSM) (24).

The aim of the present study was to evaluate the *in vitro* efficacy of exebacase on a set of S. epidermidis isolates involved in PJIs. For this purpose, we first determined exebacase MICs, then we evaluated its activity on a subset of these strains in mature biofilms in comparison to and in association with vancomycin, daptomycin, and rifampicin, commonly used antibiotics to treat PJI when multiresistant S. epidermidis strains are encountered.

## RESULTS

### Exebacase MIC.

The susceptibility to exebacase was evaluated against 19 S. epidermidis strains isolated from PJI. The MIC values ranged from 0.125 mg/L to 2 mg/L (Table 1) and were similar to values previously determined for S. epidermidis isolates using the exebacase susceptibility test medium (25). The impact of the addition of horse serum and DTT to the culture medium on the MIC values of the antibiotics tested was minimal (Fig. S1).

**TABLE 1:** Characteristics of the tested strains^*a*^

			Antibiotic susceptibility (MIC [mg/L])				Exebacase				
Strain ID	Biofilm production^*b*^	Location	Oxacillin	Rifampicin	Vancomycin	Daptomycin	MIC (mg/L)	Bactericidal effect 50 mg/L (log)	Anti-biomass effect 50 mg/L	Bactericidal effect 150 mg/L (log)	Anti-biomass effect 150 mg/L
12	None	Knee	S (<0.25)	S (<0.03)	S (1)	S (0.38)	0.125	−1.4	0%	−2.8	−63%
22	None	Knee	S (<0.25)	S (<0.03)	S (1)	S (0.38)	1	0.4	−39%	−1.3	−38%
4	Low	Knee	R (>2)	S (<0.03)	S (1)	S (0.5)	1	0.1	+49%	−1.8	−21%
10	Low	Hip	R (>2)	S (<0.03)	S (1.5)	S (0.25)	0.25	0,2	−55%	−1.7	−94%
19	Low	Hip	S (<0.25)	S (<0.03)	S (2)	S (0.5)	1	−2.5	−22%	−2.7	−40%
24	Low	Knee	R (>2)	S (<0.03)	S (1)	S (0.5)	1	0.3	−65%	0.0	−57%
33	Low	Shoulder	R (>2)	S (<0.03)	S (1)	S (0.5)	0.125	−1.8	−18%	−2.2	−79%
34	Low	Knee	R (>2)	R (>2)	S (1)	S (0.5)	0.25	0.0	−42%	−2.7	−66%
3	Moderate	Knee	R (>2)	S (<0.03)	S (1)	S (0.75)	2	−0.8	−54%	−2.6	−49%
7	Moderate	Knee	R (>2)	S (<0.03)	S (2)	S (0.19)	1	−0.6	−43%	−2.0	−76%
11	Moderate	Knee	S (<0.25)	R (>2)	S (2)	S (0.5)	0.125	−0,3	−82%	−2.2	−99%
13	Moderate	Knee	R (>2)	R (>2)	S (1)	S (0.5)	0.125	−2.0	−83%	−1.8	−86%
20	Moderate	Knee	R (>2)	R (>2)	S (1)	S (0.5)	0.25	−0.7	−54%	−1.4	−77%
25	Moderate	Hip	R (>2)	S (<0.03)	S (2)	S (0.5)	1	0.0	−51%	−1.1	−93%
32	Strong	Knee	S (<0.25)	S (<0.03)	S (1)	S (0.38)	0.5	−1.9	−58%	−2.3	−68%
39	Strong	Shoulder	S (<0.25)	S (<0.03)	S (2)	S (0.38)	2	0.1	−29%	−0.5	−32%
41	Strong	Knee	S (<0.25)	S (<0.03)	S (2)	S (0.5)	0.125	0.4	−73%	−0.7	−85%
51	Strong	Hip	S (<0.25)	S (<0.03)	S (2)	S (0.125)	1	−2.4	−57%	−2.7	−81%
52	Strong	Knee	R (>2)	R (>2)	S (1)	S (0.064)	2	0.5	−44%	−0.5	−43%

a S, susceptible; R, resistant.

b Biofilm production evaluated according to Stepanović et al. 2000.

### Exebacase activity against S. epidermidis in biofilm.

**FIG 1:**
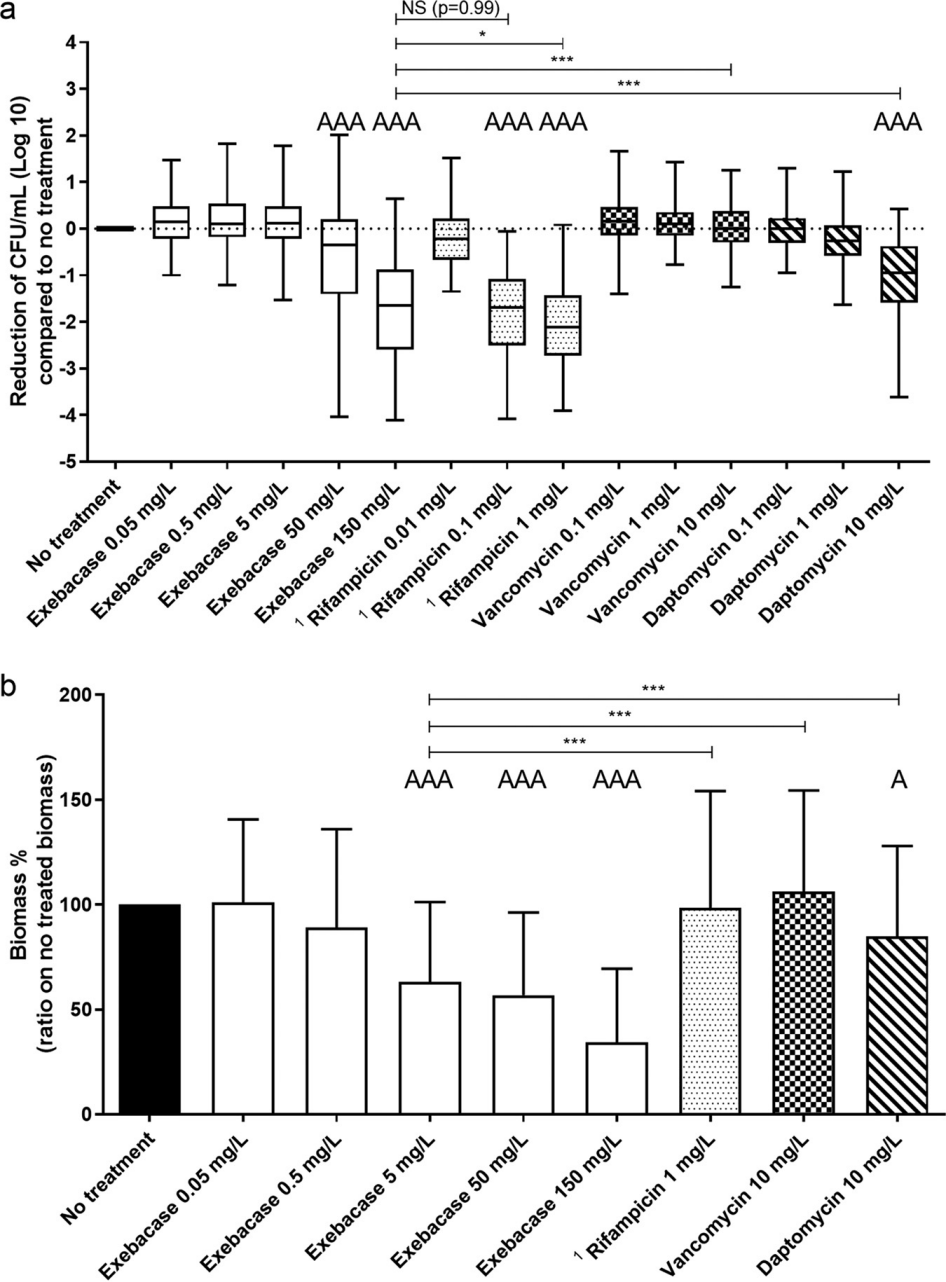
Exebacase activity in comparison to antibiotics against S. epidermidis biofilms from 19 isolates. a) Bactericidal activity: reduction of bacterial population within the preformed biofilms after 24 h of exebacase treatment at 0.05, 0.5, 5, 50, and 150 mg/L, or rifampicin at 0.01, 0.1, and 1 mg/L, vancomycin at 0.1, 1, and 10 mg/L, and daptomycin at 0.1, 1, and 10 mg/L, evaluated by enumeration of colonies on Columbia blood agar plates after serial dilutions. b) Anti-biomass activity: ratio of remaining biomass after 24 h of exebacase exposure at 0.05, 0.5, 5, 50, and 150 mg/L, rifampicin at 1 mg/L, vancomycin 10 mg/L, or daptomycin 10 mg/L, on the remaining biomass of the condition without exposition, evaluated by crystal violet staining. *, *P* < 0.05; **, *P* < 0.01; ***, *P* < 0.001; A: activity compared to no treatment (*P* < 0.05); AA: activity compared to no treatment (*P* < 0.01); AAA: activity compared to no treatment (*P* < 0.001); ^1^ the 5 rifampicin-resistant isolates were excluded.

**(i) Exebacase bactericidal activity against biofilm.** Exposure of the biofilms formed by the 19 clinical isolates to exebacase demonstrated a dose-dependent reduction in the number of viable bacteria in the biofilms compared to the untreated control, with significant mean reductions at 50 and 150 mg/L of 0.7 log and 1.7 log, respectively (p*_adj_* ≤ 0.001, Fig. 1a). Rifampicin at 1 and 0.1 mg/L, and daptomycin at 10 mg/L also showed significant mean bactericidal activity on S. epidermidis biofilms compared to the untreated control (p*_adj_* = <0.001, Fig. 1a), while no bactericidal activity was observed after exposure to vancomycin at any of the tested concentrations. At 150 mg/L exebacase demonstrated significantly greater bactericidal activity against S. epidermidis biofilms compared to daptomycin or vancomycin at 10 mg/L (p*_adj_* < 0.001), but equivalent and lower activity compared, respectively, to rifampicin at 0.1 mg/L (p*_adj_* = 0.9998, 14 strains), and 1 mg/L (p*_adj_* = 0.0148, 14 strains; Fig. 1a).

**(ii) Exebacase anti-biomass activity.** Using crystal violet staining, a significant mean decrease in biofilm biomass was observed after exebacase exposure at 5 mg/L (−37%), 50 mg/L (−43%), and 150 mg/L (−66%), in comparison to the untreated control (p*_adj_* ≤ 0.001, Fig. 1b). Daptomycin at 10 mg/L also displayed a significant mean anti-biomass activity (−15%) on S. epidermidis biofilms compared to the untreated control (p*_adj_* = 0.0314), but no anti-biomass activity was observed for rifampicin at 1 mg/L (−2%) and vancomycin at 10 mg/L (+6%). Concentrations of exebacase ≥5 mg/L had an anti-biomass effect greater than daptomycin (10 mg/L), rifampicin (1 mg/L), and vancomycin (10 mg/L; p*_adj_* < 0.001; Fig. 1b).

### Heterogeneous activity of exebacase.

We observed that anti-biofilm activity varied across tested strains. For instance, viable count reduction in the biofilm ranged from 0 to 3 log at 150 mg/L exebacase. The extent of anti-biomass activity of exebacase was also dependent on the strain and the mean decrease ranged from −21% to −99%. Similar results were obtained with exebacase at 50 mg/L with the reduction in viable count in the biofilm ranging from +0.4 to −2.5 log and anti-biomass activity ranging from +49 to −83%. The ANOVA test showed that 11.1% of the observed variability in bacterial killing and 7.5% of the observed variability in the anti-biomass effect were due to the strain (Fig. S2).

Exebacase anti-biomass and bactericidal activity on biofilm were not correlated with the biofilm production phenotype (Spearman correlation, p*_adj_* = 1.000). Exebacase activity also did not correlate with exebacase MIC values (Spearman correlation, p*_adj_* = 0.272 (rho = −0.069) and p*_adj_* = 0.144 (rho = −0.059) for anti-biomass and bactericidal activity, respectively). Bactericidal activity of exebacase correlated with rifampicin resistance phenotype (Spearman correlation, p*_adj_* < 0.001 [rho = 0.143]) with exebacase appearing to be more active against rifampicin susceptible isolates. Finally, anti-biomass activity of exebacase correlated with rifampicin resistance phenotype (p*_adj_* = 0.016 [rho = 0.103]), with exebacase appearing to be more active against rifampicin-resistant isolates. No correlation was observed for oxacillin resistance and bactericidal or anti-biomass activity (p*_adj_* = 0.248 and p*_adj_* = 1.000).

### Activity of the exebacase used in addition to antibiotics on S. epidermidis biofilms.

**FIG 2:**
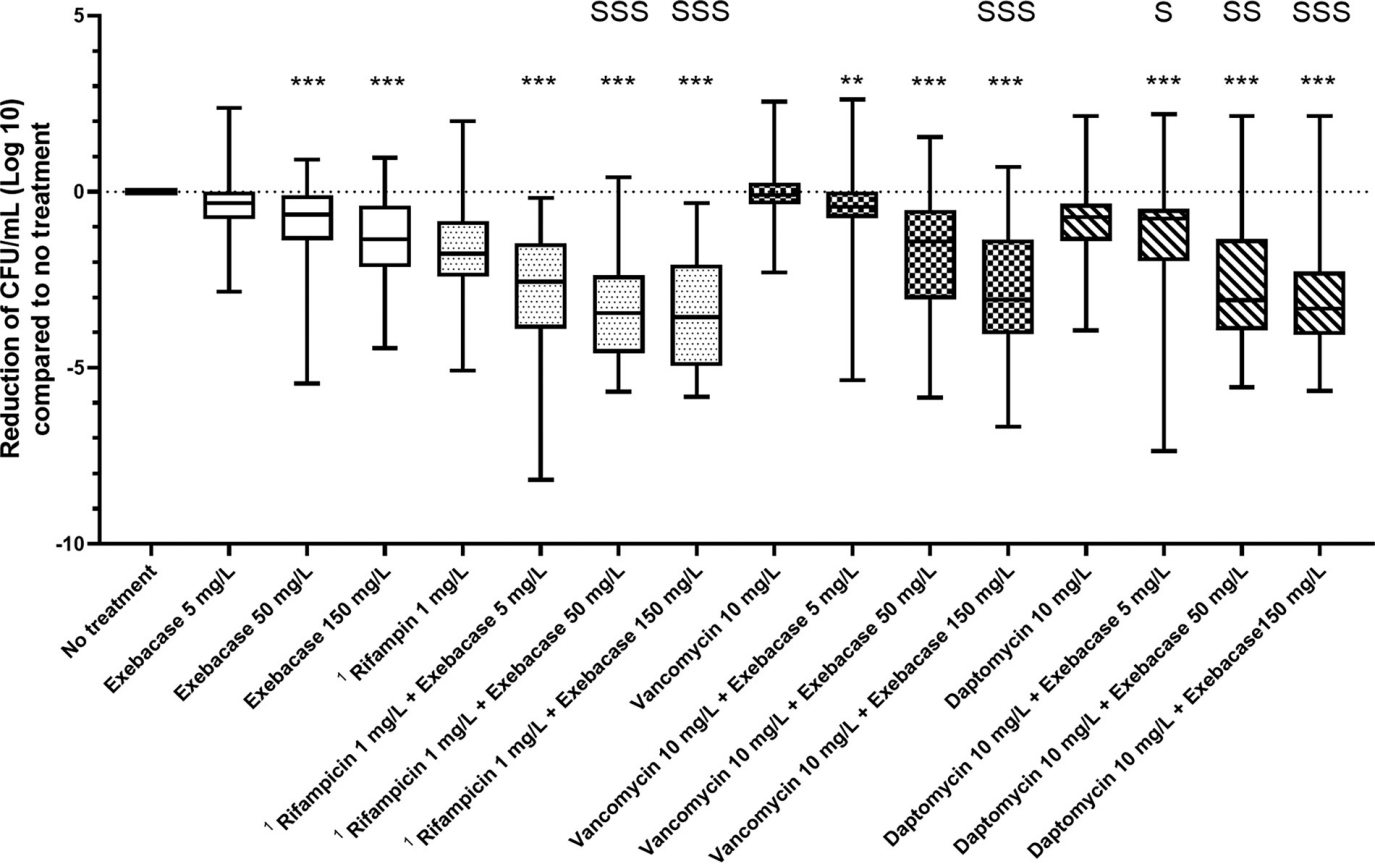
Anti-biofilm activity of exebacase in addition to antibiotics compared to exebacase and antibiotics alone against S. epidermidis biofilms from 19 isolates. Reduction of living bacteria inside the biofilms after 24 h of exebacase exposure at 5, 50, and 150 mg/L, and/or rifampicin at 1 mg/L, vancomycin at 10 mg/L, and daptomycin at 10 mg/L, evaluated by enumeration of colonies on Columbia blood agar plates after serial dilutions. *, *P* < 0.05; **, *P* < 0.01; ***, *P* < 0.001; S: synergy *P* < 0.05; SS: synergy *P* < 0.01; SSS: synergy *P* < 0.001, ^1^ the 5 rifampicin-resistant isolates were excluded.

**FIG 3:**
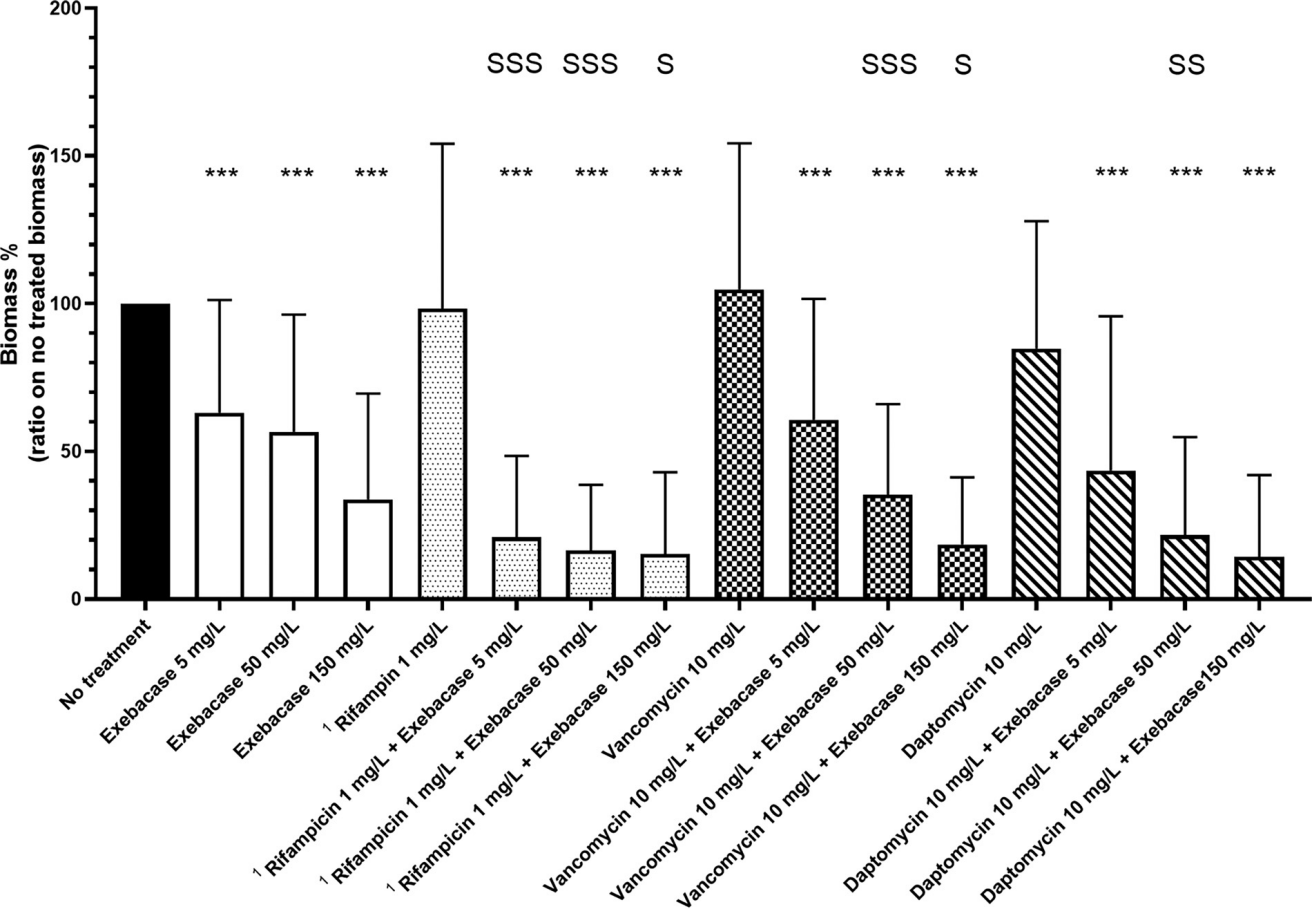
Anti-biomass activity of exebacase in addition to antbiotics compared to exebacase and antibiotics alone against S. epidermidis biofilms from 19 isolates. Remaining biomass after 24 h of exebacase exposure at 5, 50, and 150 mg/L, and/or rifampicin at 1 mg/L, vancomycin at 10 mg/L, and daptomycin 10 mg/L, evaluated by crystal violet staining. *, *P* < 0.05; **, *P* < 0.01; ***, *P* < 0.001; S: synergy *P* < 0.05; SS: synergy *P* < 0.01; SSS: synergy *P* < 0.001, ^1^ the 5 rifampicin-resistant isolates were excluded.

After 24 h of treatment, significant bactericidal and anti-biomass effects of the use of exebacase in addition to antibiotics were observed for all exebacase concentrations (p*_adj_* < 0.001), except for the bactericidal activity of vancomycin (1 mg/L) and exebacase (5 mg/L) (p*_adj_*: 0.00938) (Figs. 2 and 3). We determined if each specific association is synergistic, additive or antagonistic (please see statistical analysis section for definitions).

### Exebacase used in addition to rifampicin.

A synergistic bactericidal effect was observed for the exebacase (50 and 150 mg/L) and rifampicin (1 mg/L) (p*_adj_* < 0.001) (Fig. 2) while it was only additive at 5 mg/L of exebacase (p*_adj_* = 0.1844). Significant synergistic anti-biomass effects were shown for all three tested exebacase concentrations (p*_adj_* < 0.001 for 5 and 50 mg/L, and 0.041 for 150 mg/L). The exebacase used in addition to rifampicin led to a decrease of the bacterial count of 3.5 log and of 84% of the biomass, 3.5 log and 83%, and 2.7 log and 79% for 150, 50, and 5 mg/L exebacase concentrations, respectively (Figs. 2 and 3).

### Exebacase used in addition to vancomycin.

A synergistic bactericidal effect was observed for the exebacase (150 mg/L) in addition to vancomycin (10 mg/L) (p*_adj_* < 0.001), while at 5 and 50 mg/L of exebacase the effect was only additive (p*_adj_* = 0.13 and 0.056, respectively) (Fig. 2). Synergistic anti-biomass effects were observed for 50 and 150 mg/L exebacase concentrations (p*_adj_* < 0.001 and 0.017, respectively). At 5 mg/L, the use of exebacase in addition to vancomycin had an additive effect (p*_adj_* = 0.40). The exebacase used in addition to vancomycin led to a decrease of the inoculum of 2.8 log and 81% of the biomass, 1.9 log and 66%, and 0.6 log and 40% for 150, 50, and 5 mg/L exebacase concentrations, respectively (Figs. 2 and 3).

### Exebacase used in addition to daptomycin.

Synergistic bactericidal effects were observed for all concentrations of exebacase used in addition to daptomycin (10 mg/L)] (p*_adj_* = 0.014, 0.002, and <0.001 for 5, 50, and 150 mg/L, respectively) (Fig. 2). A synergistic anti-biomass effect was found for the exebacase (50 mg/L) used in addition to daptomycin (10 mg/L) (p*_adj_* = 0.0098), while the effects were additive at 5 and 150 mg/L (p*_adj_* = 0.45 and 0.46, respectively). The exebacase used in addition to daptomycin led to a decrease of the inoculum of 3.1 log and 85% of the biomass, 2.7 log and 78%, and 1.4 log and 56% for 150, 50, and 5 mg/L exebacase concentrations, respectively (Figs. 2 and 3).

## DISCUSSION

In the present study, we assessed exebacase activity against biofilms from 19 S. epidermidis isolates from PJI, by measuring both bactericidal and anti-biomass activity. Exebacase displayed significant anti-biomass and bactericidal activity against S. epidermidis biofilms as well as synergistic effects in addition to rifampicin, vancomycin, and daptomycin.

Biofilms are frequently investigated using 96-well microtiter plates, but this model often lacks reproducibility due to aggressive washing (26). In this study, we used BiofilmCare method, which allows for nonaggressive washing, and provides more reproducible results (27). Rather than just investigating anti-biofilm activity by only measuring viable bacteria within biofilm after treatment as is often done, we also studied the disruption of the biomass itself (28). Both these activities are of interest for biofilm associated infections, such as BJI, as they play a role in restoring activity of antibiotics against biofilms when used together. This model was sufficient for biofilm screening, however, the use of standard culture media such as TSB did not mimic the microenvironment of an infected bone or prosthetic joint infection. The adjunction of synovial fluids for further studies may improve its performances in mimicking articular micro-environment (29).

Exebacase was used at five concentrations (0.05, 0.5, 5, 50, and 150 mg/L), corresponding to approximately 0.1, 1 and 10 MICs, and two concentrations framing concentrations used by Ferry et al. during local administration (24). For tested antibiotics, 3 concentrations (1×, 10× and 100×) were used in order to be close to the intra-osseous concentrations and to MICs breakpoints (30). For Rifampicin, as breakpoints are low, lower concentrations than for vancomycin and daptomycin were chosen.

Several studies showed that exebacase exhibited *in vitro* activity against planktonic S. epidermidis strains (20), as well as against S. epidermidis biofilms (16). This study confirmed these activities, but anti-biofilm activity was only observed at supra-MICs, while Schuch et al. demonstrated that exebacase was able to disrupt S. aureus and mixed S. aureus – S. epidermidis biofilms entirely at sub-MICs (16). The reason for this discrepancy could be the origin of the isolates which, in this study, were collected from patients with chronic infections, resulting in potential host-adaptation and modified biofilms which may have impaired exebacase activity (31). For further studies, it could be interesting to use a collection mixing isolates from this study and from Schuch et al. study to decipher the role of strain characteristics during exebacase treatment. As shown here for exebacase, rifampicin and daptomycin also exhibited anti-biofilm activities only at supra-MICs concentrations, in agreement with other studies (32, 33). The anti-biofilm and anti-biomass activities were both concentration- and strain-dependent, but anti-biomass activity was always observed at lower concentrations, suggesting that exebacase needed to disrupt biofilm first, thanks to a potential proteolytic activity, to allow its penetration inside the biofilm to exert its bactericidal activity. Specific effects of exebacase on biofilm matrix and relationship with matrix composition need to be investigated in further studies.

Based on the use of this set of clinical isolates, our data highlighted strain-dependent behavior toward anti-biofilm activity of exebacase. We showed that exebacase activity was not correlated with biofilm formation phenotype, but this heterogeneous behavior may be related to the biofilm composition. Staphylococcal biofilms are composed of PIA, proteins, and extracellular DNA, and the heterogeneous proportion of each of these elements may explain the heterogeneous response to exebacase (9–11). Further studies are needed to identify the genetic/physiological basis for this heterogeneity. For instance, S. epidermidis strains with deletions of genes encoding matrix, proteins, PIA and extracellular DNA synthesis would be good candidates for future investigations (34, 35). The use of confocal microscopy and specific staining on biofilms would allow the exploration of the composition of the biofilm and thus to match the composition with the heterogeneous responses obtained in our study. Another hypothesis is related to the mechanism of action of exebacase, which cleaves the interpeptide bridge of peptidoglycan (15, 20, 36–38). Compared to S. aureus, the interpeptide bridge structure in S. epidermidis is variable (39, 40), thus changes in the amino acid composition could lead to a change of activity of exebacase, as observed with lysostaphin (41), another cell wall hydrolase, or to a decreased exebacase affinity, as exebacase recognizes and binds to a specific interpeptide bridge pattern (42). However, it would be expected that modifications of the interpeptide bridge of peptidoglycan would lead to altered exebacase MIC values and because no correlation was found between exebacase MIC values and antibiofilm activity, this hypothesis seems unlikely.

Exebacase activity was compared to that of rifampicin, vancomycin and daptomycin, antibiotics frequently used in PJI treatment. While vancomycin has no activity against biofilms (28), rifampin and daptomycin show good diffusion within bones and biofilms (28, 43, 44), and they are frequently used in empirical antibiotic treatment following bone and joint surgery. Exebacase’s bactericidal activity against S. epidermis biofilms was better than vancomycin, and daptomycin and equivalent to or lower than rifampicin. In addition, exebacase showed better anti-biomass effect than any of these antibiotics. This suggests that exebacase is able to disrupt biomass more effectively than rifampicin and daptomycin.

It has been shown that exebacase can synergistically improve activities of daptomycin and vancomycin against planktonic S. aureus (15, 23, 25, 45, 46). Here, we demonstrated that exebacase also exhibits synergistic antibiofilm and anti-biomass effects with rifampicin, vancomycin and daptomycin against S. epidermidis biofilms. Exebacase-rifampicin showed the best antibacterial effects against S. epidermidis grown in a biofilm with 115% and 27% reductions, respectively, compared to each drug alone. The treatment in addition to vancomycin resulted in 100% and 23% improvements, whereas for daptomycin 121% and 29% improvements were observed for antibiofilm and anti-biomass activities, respectively.

A likely explanation for the synergy is that exebacase restores the access of the antibiotics to their targets within the biofilm, thereby dramatically improving their activity. It must be noticed that the definition used in the present study for “synergy” was stringent, notably for the highest concentration of exebacase. Indeed, as the effect of the agents together had to be greater than the sum of the effect of each molecule alone to define a synergistic effect, if the activity of a single molecule is strong, the demonstration of synergy is challenging because the limit of enumeration or crystal violet detection for our model are reached. This may explain the apparent absence of anti-biomass synergy between exebacase (150 mg/L) and daptomycin (10 mg/L) and the poorer significance obtained with higher concentrations of exebacase (150 mg/L). Conversely, the apparent absence of synergy at low concentration of exebacase (5 mg/L) may be related to a too low activity of exebacase at this concentration not allowing for sufficient destruction of the biofilm to restore the access of antibiotics to their targets. These *in vitro* data suggest that using exebacase in addition to traditional antibiotics to treat PJI could improve biofilm removal and thereby improve recovery in patients. Beyond biofilms, small colony variants and internalization of bacteria in osteoblasts are also involved in PJIs, and further investigations are needed to assess the exebacase activity on these bacterial reservoirs for S. epidermidis. Of note, Schuch et al. have shown that exebacase activity was not impacted by small colony variants of S. aureus (16).

In this study, exebacase antibiofilm activity was correlated with rifampicin susceptibility phenotypes; however, rifampicin resistance was responsible for only a fraction of the variability observed for biofilm bactericidal and anti-biomass activity. Rifampicin-resistant isolates tended to be less susceptible to exebacase biofilm bactericidal activity. In contrast, rifampicin-resistant isolates tend to be more susceptible to exebacase anti-biomass activity. As anti-biomass activity was dependent on biofilm composition, it would be interesting to compare biofilm composition of rifampicin-resistant and -susceptible isolates. The strains tested in this study were only resistant to oxacillin and/or rifampicin, which were, respectively, due to the acquisition of PBP2a (47) or mutations in the *rpoB* gene (48). None of these have an impact on peptidoglycan composition, consistent with the similar activity of exebacase against these isolates. In contrast, vancomycin and daptomycin resistance are due to peptidoglycan modifications (39, 49) that in turn may affect the activity of exebacase, although Gilmer et al. showed that vancomycin and daptomycin resistance in S. aureus did not affect the susceptibility to exebacase (20). Such strains were not detected in the S. epidermidis clinical strains collected at the *Hôpital de la Croix-Rousse* and could not be included in our study.

Exebacase may be used in addition to antibiotics for the management of S. epidermidis biofilm associated infections, such as PJIs. It presents a strong *in vitro* bactericidal and anti-biomass activity on S. epidermidis biofilms, potentiated when combined with antibiotics.

## MATERIALS AND METHODS

### Strains.

This study was performed on S. epidermidis clinical strains collected at the *Hôpital de la Croix-Rousse* (*Hospices Civils de Lyon*, France) between 2015 and 2018 from patients with confirmed PJI (i.e., cases in which at least 2 samples were positive, according to the Musculoskeletal Infection Society criteria) (50). Nineteen strains were selected based on their biofilm formation phenotype according to the Stepanovic’s method (51) and their antibiotic resistance phenotypes (Table 1).

### MIC assessments.

Exebacase MICs against S. epidermidis were determined using the Clinical and Laboratory Standards Institute (CLSI)-approved medium for antimicrobial susceptibility testing of S. aureus (48). The medium is comprised of cation-adjusted Mueller-Hinton broth (CAMHB, BD BBLTM) with 25% horse serum (Sigma-Aldrich) and 0.5 mM DTT (Dithiothreitol, Sigma-Aldrich) (CAMHB-HSD) (52). MICs were assessed after 18 h of incubation at 35°C (+/−2°C), as previously described using S. epidermidis (25). S. aureus ATCC 29213 and Enterococcus faecalis ATCC 29212 were used as quality controls. The MICs of antibiotics were also measured in this media to establish the influence on antimicrobial activity.

### Evaluation of activity against S. epidermidis biofilms.

The evaluation of exebacase activity on mature biofilms was performed using the BiofilmCare method (27). Briefly, biofilms were formed in duplicate using two 96-well microplates for 24 h in TSB glucose 1% (BD BBLTM), then steam-washed for 40 min. Preformed biofilms were then treated at 35°C (+/−2°C) for 24 h with exebacase (0.05, 0.5, 5, 50, and 150 mg/L when used alone, 5, 50, and 150 mg/L when used in addition to antibiotics), and/or vancomycin (0.1, 1, and 10 mg/L for bactericidal activity on biofilm when used alone, 10 mg/L otherwise), rifampicin (0.01, 0.1, and 1 mg/L for bactericidal activity on biofilm when used alone, 1 mg/L otherwise), or daptomycin (0.1, 1, and 10 mg/L for bactericidal activity on biofilm when used alone, 10 mg/L otherwise) in CAMHB-HSD. For daptomycin the medium was supplemented with calcium (final concentration: 50 mg/L) (53). Untreated controls (incubation in CAMHB-HSD alone) were included in every experiment. For the first 96-well plate, the medium was removed, and remaining biofilms were again steam-washed and then resuspended by scratching in 1× PBS (Dulbecco's phosphate buffer saline, Thermo Fisher) followed by sonication for 10 min (40 Hz, BactoSonic Bandelin), and serial dilutions were then plated on Columbia blood agar plates (bioMérieux) to enumerate the remaining viable bacteria. For the second 96-well plate mirroring the first one, biomass reduction (extracellular matrix, live and dead bacteria) was evaluated by crystal violet staining. After exposure to exebacase +/-antibiotics, the remaining biofilm was washed using the BiofilmCare method and covered with crystal violet (ELITech Group), incubated for 10 min, and then again steam-washed for 60 min. Finally, 200 µL of glacial acetic acid at 33% (vol/vol) was added to dried plates to dissolve the remaining crystal violet. Absorbance (OD_590_) was then measured using an automatic microplate reader (Infinite M Nano +, Tecan).

### Statistical analysis.

Statistical analysis was performed using the R software v3.6.3 (R Foundation for Statistical Computing) (54). The significance level used was *P* < 0.05 regardless of the test. All tests were two-sided. For statistical analysis, 6 values were considered per strain, 19 strains per condition, except for rifampicin for which 14 stains were considered (the 5 remaining strains were resistant to rifampicin). ANOVA followed by Dunnett’s correction was used to compare the activities of exebacase or antibiotics to the absence of exposure to these molecules. ANOVA followed by Dunnett’s correction was also used to compare antibiotic activities to exebacase. Paired *t* test followed by Bonferroni correction were used to determine synergy between exebacase and antibiotics. Synergy is defined as a significantly higher effect of the association in comparison to the sum of the effects of each treatment alone. If the effect of the association is equal to the sum of the effects of each treatment alone, additivity is observed. Otherwise, it is considered antagonism. Correlations were performed using Spearman’s rank correlation followed by Bonferroni correction.
