# Supplementary material for: Activity of Exebacase (CF-301) against Biofilms Formed by Staphylococcus epidermidis Strains Isolated from Prosthetic Joint Infections
Source: Antimicrob Agents Chemother. 2022 Jul 11;66(8):e00588-22. doi: 10.1128/aac.00588-22 (PMC9380561; doi:10.1128/aac.00588-22)
Supplement: Supplemental file 1 — Figs. S1 and S2. Download aac.00588-22-s0001.pdf, PDF file, 0.5 MB [file aac.00588-22-s0001.pdf]

Supplementary data

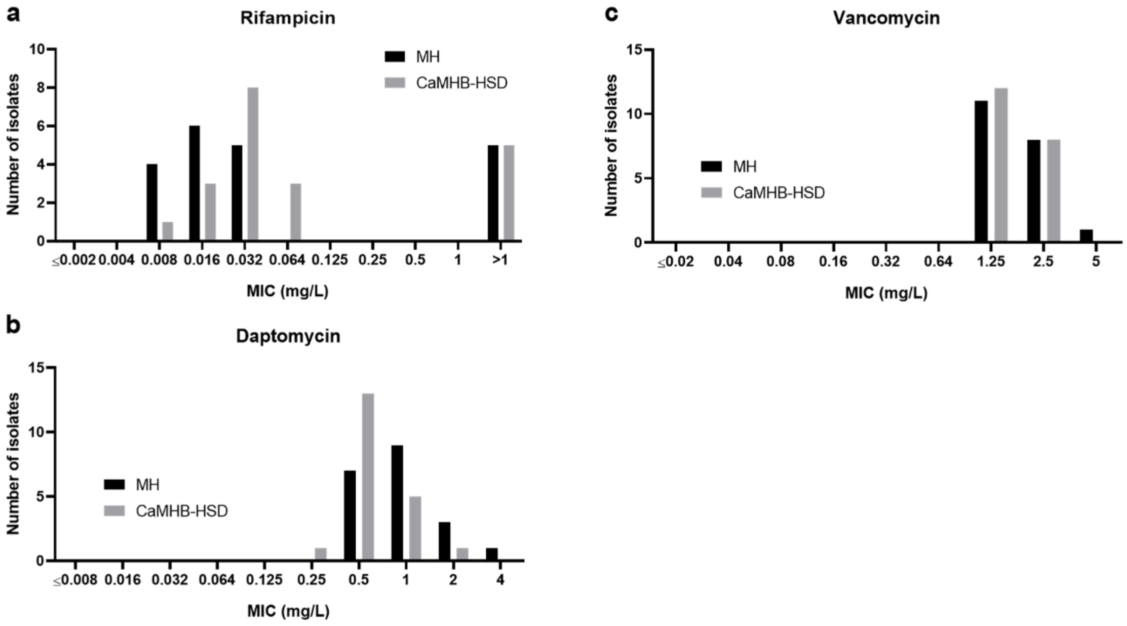

Figure S1: MIC for antibiotics against the 19 tested *S. epidermidis* strains according to the culture media.

MICs were determined by broth microdilution in Mueller-Hinton (MH) or CAMHB-HSD medium for rifampicin, vancomycin and in MH or CAMHB-HSD medium supplemented with calcium (final concentration: 50 mg/L) for daptomycin.

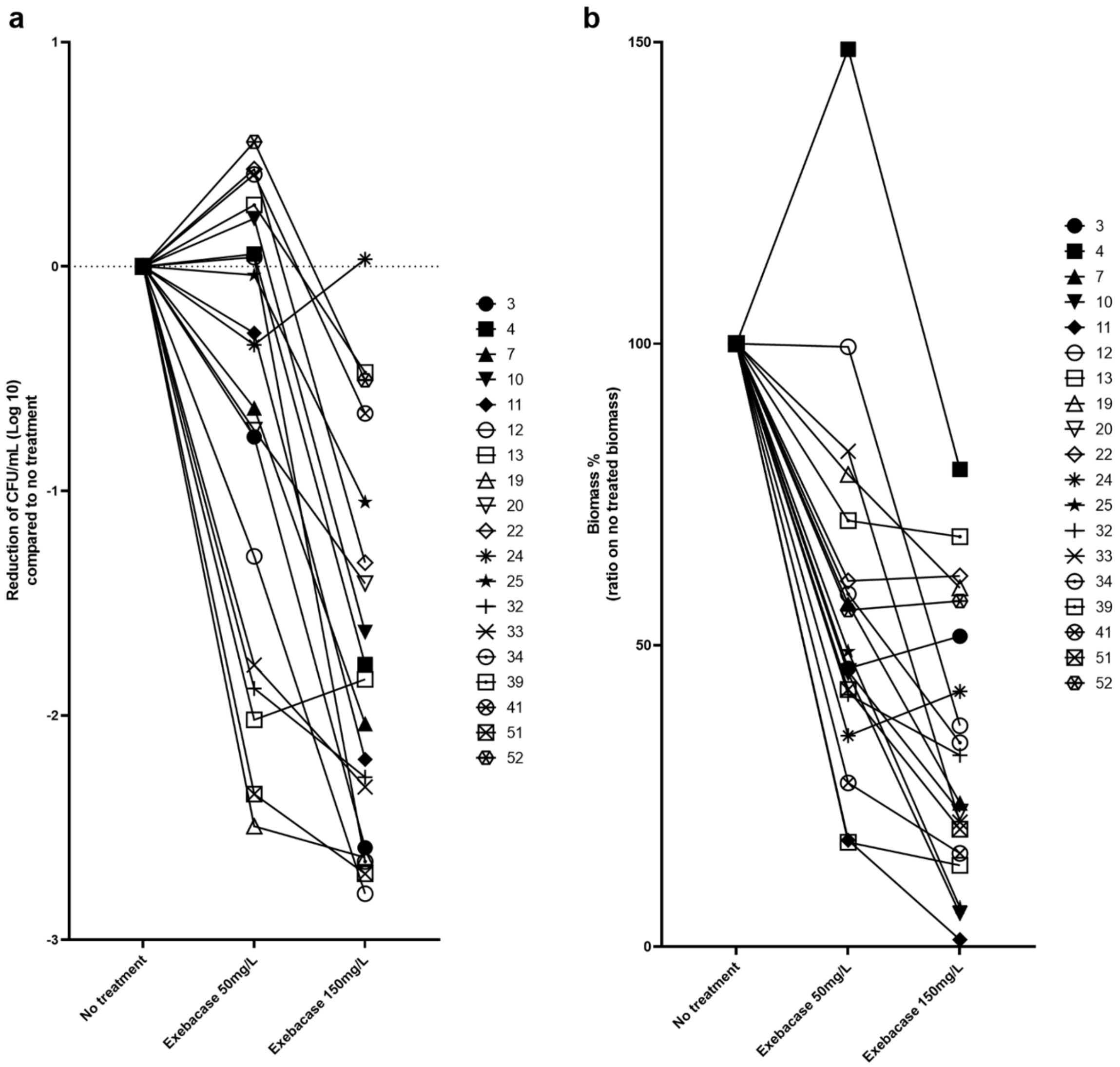

Figure S2: Exebacase anti-biofilm and anti-biomass activity on 19 tested *S epidermidis* strains by strain.

a) Reduction of living bacteria inside the biofilms after 24 hours of exebacase exposure at 50, and 150 mg/L, evaluated by enumeration of colonies on Columbia blood agar plates after serial dilutions. b) Remaining biomass after 24 hours of exebacase exposure at 50, and 150 mg/L, evaluated by Crystal Violet staining.
